# Supplementary material for: Topographic Guidance in Melt-Electrowritten Tubular Scaffolds Enhances Engineered Kidney Tubule Performance
Source: Front Bioeng Biotechnol. 2021 Jan 18;8:617364. doi: 10.3389/fbioe.2020.617364 (PMC7848123; doi:10.3389/fbioe.2020.617364)
Supplement: Supplementary Figure 1 — (A) Schematic of the melt electrowriting set-up, showing the collection of thin electrified polymer jets onto a computer controlled, rotating and translating mandrel. (B) Representation of the mandrel velocities, translational (Vtrans), tangential (Vtang), rotational (Vrot), as well as winding angle (α), coil angle (⊖), tube length, pitch and mandrel perimeter (r) in the mandrel surface during fiber collection. Winding angle was engineered by the ratio between Vtang and Vtrans at constant fiber length. [file Table_1.DOCX]

**Table S1**.

| **Composition** | **Water contact angle** | |
| --- | --- | --- |
| PCL  (close to hydrophobic) | 93.07 ± 3.87° | **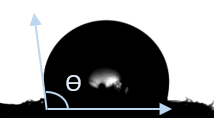** |
| L-DOPA coated PCL  (close to absolute wetting) | 0.5 ± 1.87° | **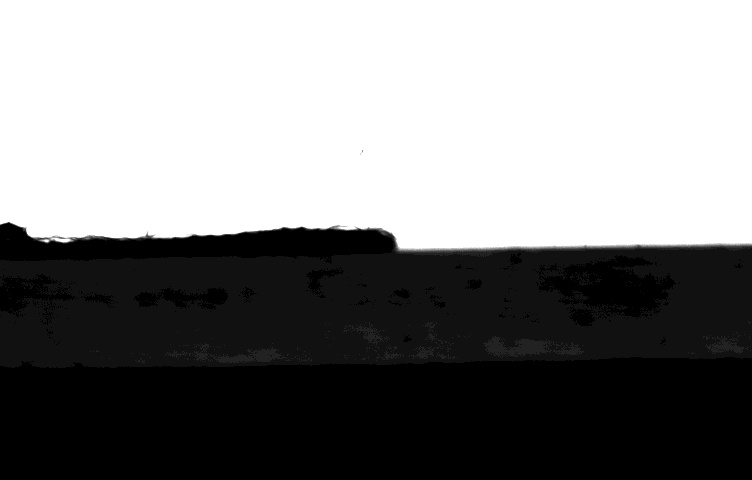** |

**
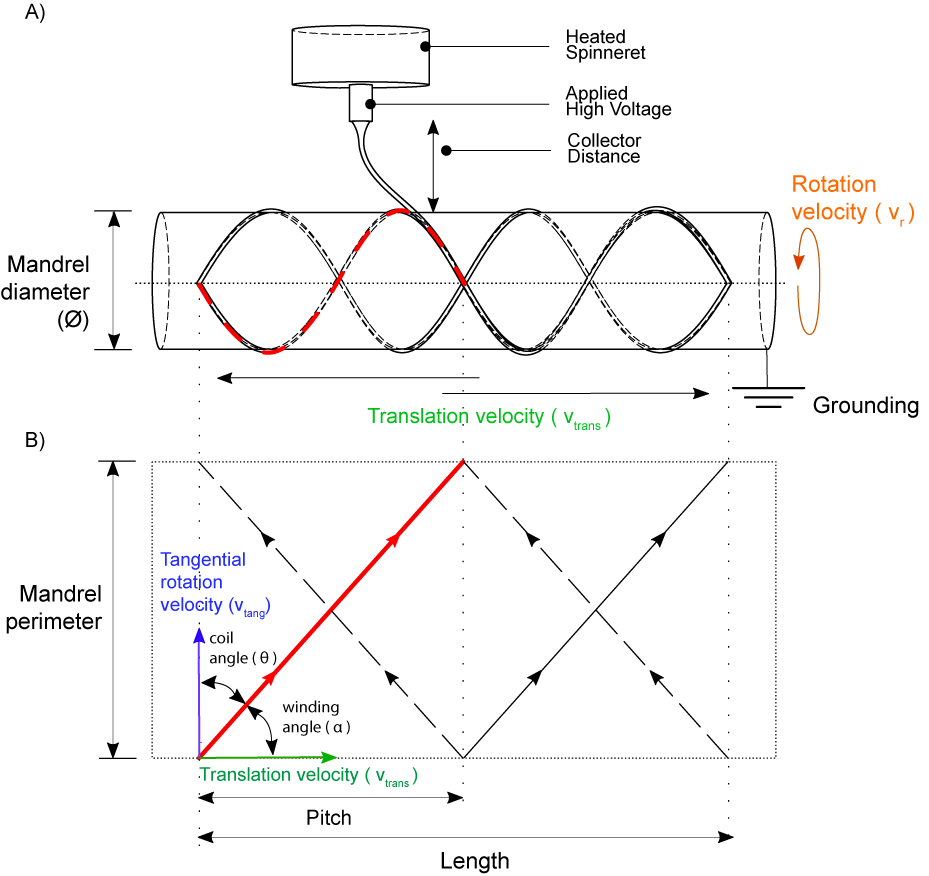
Figure S1.**

**
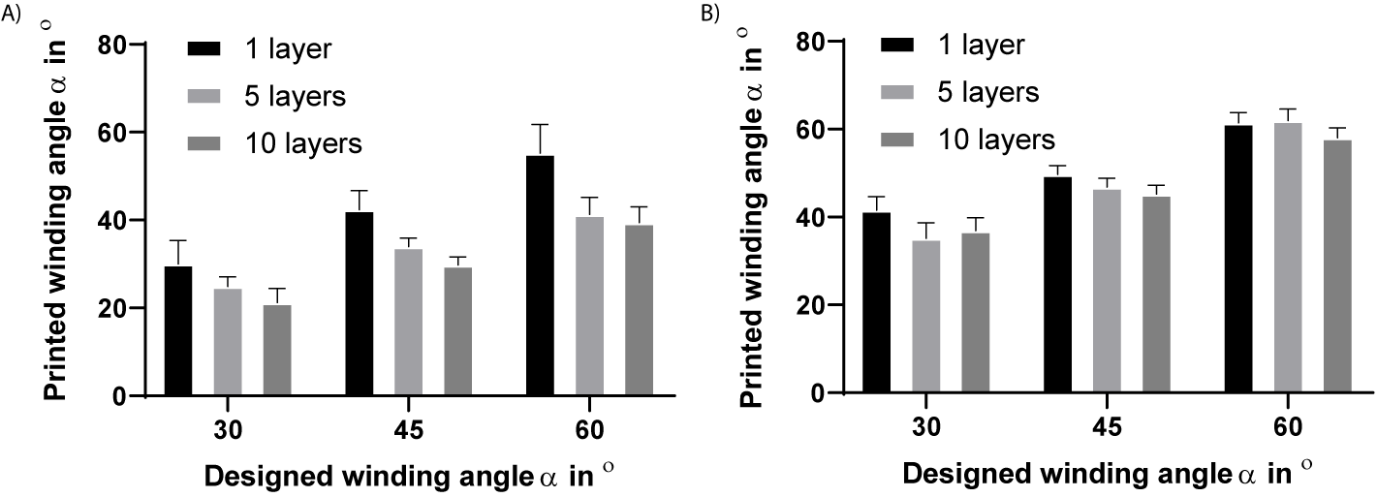
**

**Figure S2.
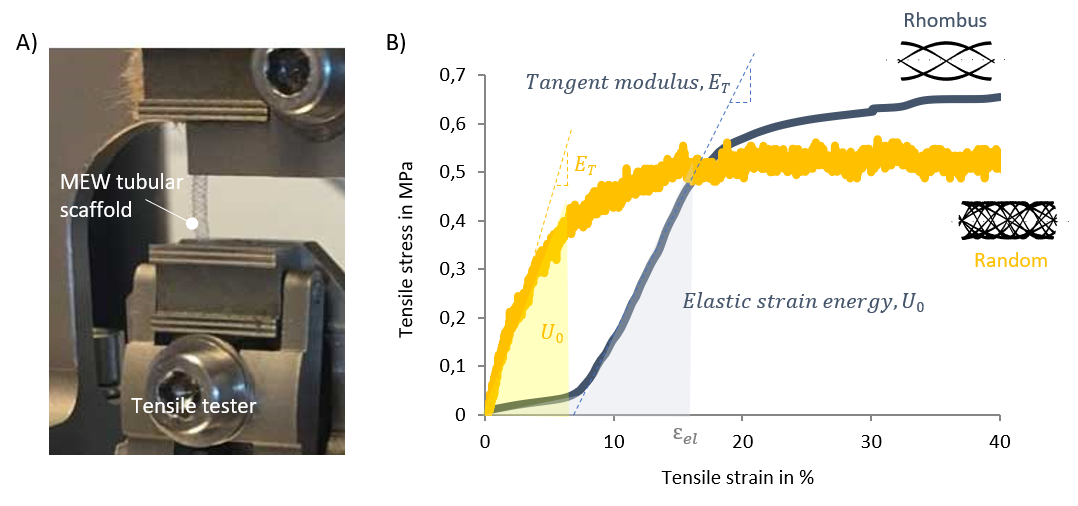
Figure S3**

**
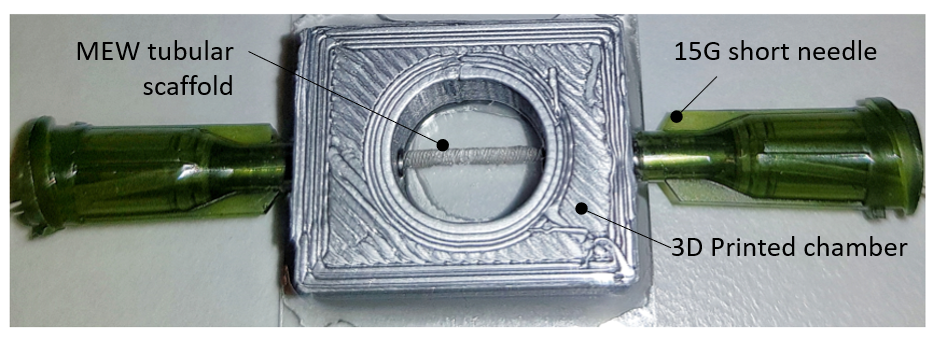
Figure S4.**
